# Lectin from green speckled lentil seeds (*Lens culinaris*) triggered apoptosis in nasopharyngeal carcinoma cell lines

**DOI:** 10.1186/s13020-015-0057-6

**Published:** 2015-09-08

**Authors:** Yau Sang Chan, Huimin Yu, Lixin Xia, Tzi Bun Ng

**Affiliations:** State Key Laboratory of Respiratory Disease for Allergy at Shenzhen University, School of Medicine, Shenzhen University, Nanhai Ave 3688, 518060 Shenzhen, Guangdong People’s Republic of China; School of Biomedical Sciences, Lo Kwee Seong Integrated Biomedical Sciences Building, The Chinese University of Hong Kong, Shatin, New Territories Hong Kong, People’s Republic of China

## Abstract

**Background:**

The green speckled lentil seed (*Lens culinaris*) lectin (GSLL) exhibits hemagglutinating activity, and possesses some properties distinct from those of other lentil lectins (e.g., molecular size, biological activities) that deserve further investigation. This study aims to investigate the basic properties (e.g., molecular size, amino acid sequence, sugar specificity) and biological activities (e.g., antiproliferative activity) of GSLL.

**Methods:**

GSLL was purified by successive fractionation on SP-Sepharose, Affi-gel blue gel, Mono Q, and Superdex 75. The biochemical properties of GSLL were investigated by SDS-PAGE, mass spectrometry, N-terminal amino acid sequencing, and sugar inhibition tests. For the biological activities, purified lyophilized GSLL was sterilized, adjusted to concentrations from 1 to 0 mg/mL (by twofold serial dilution) in Dulbecco’s modified Eagle’s medium with fetal bovine serum, and examined by using the MTT assay, flow cytometry, and western blotting after treatment of nasopharyngeal carcinoma CNE1 and CNE2 cell lines with the lectin.

**Results:**

GSLL appeared as a 21-kDa band in non-reducing SDS-PAGE. It was composed of two subunits with molecular sizes of 17 and ~4 kDa. It exhibited specificity in binding to glucose and mannose, as well as glucosides and mannosides. Mass spectrometry and N-terminal amino acid sequencing revealed similarity of GSLL to *L. culinaris* lectin (LcL), especially higher coverage of the β-chain of LcL. A 48-h treatment with GSLL exerted antiproliferative effects on nasopharyngeal carcinoma CNE1 and CNE2 cell lines with significant inhibition at 0.125 mg/mL (*P* < 0.001) and 1 mg/mL (*P* = 0.004), respectively, and these effects were attenuated in the presence of glucose and mannose. GSLL induced apoptosis in nasopharyngeal carcinoma CNE1 cells, with detectable phosphatidylserine externalization, mitochondrial depolarization, and cell cycle arrest. Western blot analysis suggested that GSLL triggered the extrinsic apoptotic pathway involving caspase 3, 8, and 9 in CNE1 cells.

**Conclusion:**

GSLL possessed some different properties from LcL (e.g., lower pI), and increased caspase 3, 8, and 9 activity in CNE1 cells.

## Background

Lentil (*Lens culinaris*) seeds contain high contents of proteins (~25 % dry weight) and minerals (Zn, Fe, Ca, Mn, etc.) [1, 2]. They are also rich in dietary fiber, which helps the digestive tract to function under good conditions [3], have high contents of folate and magnesium that reduce the risk of coronary heart disease [4], and possess high levels of iron [5]. In Chinese medicine, lentil seeds benefit the *spleen* (*pi*) and *stomach* (*wei*), and relieve *dampness* (*shi*) of the body [6, 7].

Lentil seeds contain a carbohydrate-binding protein, *L. culinaris* lectin (LcL) or hemagglutinin (LcH) [8]. The sequence and structure of LcLs have been elucidated [9], and they bind to glucose, mannose, and some glucose- and mannose-containing sugars, as well as to α-d-mannopyranosyl and α-d-glucopyranosyl residues of glycoproteins and glycolipids [10]. LcLs can bind to specific carbohydrate groups of glycoproteins on cell surfaces, thus allowing studies on differences in the levels of glycoproteins in different cell types [11, 12]. They are also used in affinity chromatography for detection of glucose- and mannose-containing biomarkers, e.g., LcLs can bind to α-fetoprotein in the blood for diagnosis of hepatocellular carcinoma [13, 14].

Lentils are differentiated into many different cultivars worldwide, such as the green speckled lentil in the United States. Although not manufactured in bulk quantities, the product of edible dry green speckled lentil seeds can be found in a number of supermarkets. Similar to other lentil seeds, green speckled lentil seeds contained a lectin that exhibited hemagglutinating activity [15]. Although LcLs have been well studied, little information is available on lentil lectins from other cultivars. Different cultivars of lentil seeds may generate lectins with variable activities. For example, the lectin from the extralong autumn purple bean cultivar (*Phaseolus vulgaris*) had prominent antiproliferative activity on hepatoma HepG2 cells [16]. Meanwhile, the lectin from the brown kidney bean cultivar (*P. vulgaris*) inhibited breast cancer MCF7 cells and nasopharyngeal carcinoma CNE1 cells more strongly than it inhibited HepG2 cells [17], and their counterpart from an Indian cultivar (*P. vulgaris*) was devoid of anticancer activity [18].

This study aims to investigate the basic properties (e.g., molecular size, amino acid sequence, sugar specificity) and biological activities (e.g., antiproliferative activity) of the green speckled lentil seed lectin (GSLL).

## Methods

### Purification

Green speckled lentil seeds were purchased from a local supermarket in Shatin, New Territories, Hong Kong. The seeds were authenticated using a DNA analysis method, which was carried out by Prof. Shiu-Ying Hu, Honorary Professor of Chinese Medicine, The Chinese University of Hong Kong. The seeds (80 g) were soaked in 10 mM Tris–HCl buffer (pH 7.6) overnight, homogenized in a blender, and centrifuged twice at 30,000×*g* and 4 °C for 25 min in an Avanti J-E High Speed centrifuge (Beckman Coulter, USA). The 800-mL supernatant obtained constituted a crude extract of the seeds. This extract was loaded onto an SP-Sepharose column (Code: 17-0729-04; GE Healthcare, USA) (5 cm × 15 cm) previously pre-equilibrated with the same buffer. The unbound fraction containing GSLL was collected and loaded onto an Affi-gel blue gel column (Code: 1537301; Bio-Rad, USA) (5 cm × 15 cm). The unbound materials were removed by washing with the buffer. The adsorbed materials were eluted with 1 M NaCl in 10 mM Tris–HCl buffer (pH 7.6) and collected as the bound fraction. The bound fraction was dialyzed against double-distilled water, and lyophilized into powder form.

The powder was resuspended into a 15 mg/mL protein solution in 10 mM Tris–HCl buffer (pH 7.6), and injected into a Mono Q 5/50 GL column (Code: 17-5166-01; GE Healthcare) for fast protein liquid chromatography (FPLC) with an AKTA purifier (Code: 28-4062-64; GE Healthcare). An increasing NaCl concentration was utilized to elute the column. Fractions of the first adsorbed peak corresponding to the lectin were collected, dialyzed, and lyophilized.

The powder was resuspended into a 5 mg/mL protein solution in 10 mM Tris–HCl buffer (pH 7.6), and loaded onto a Superdex 75 10/300 GL column (Code: 17-5174-01; GE Healthcare) for size-exclusion FPLC with the AKTA purifier. The last peak eluted at the 37th minute contained purified GSLL [16].

### Biochemical property tests

#### Sodium dodecyl sulfate–polyacrylamide gel electrophoresis (SDS-PAGE)

Loading buffer with (for reducing SDS-PAGE) or without (for non-reducing SDS-PAGE) β-mercaptoethanol was added to the fractions obtained at different steps during the purification of GSLL. The samples were loaded onto a polyacrylamide gel (15 % running gel; 5 % stacking gel), and subjected to SDS-PAGE at a constant voltage of 120 V for 70 min. The gel was stained with Coomassie Brilliant Blue for 1 h, and destained with 10 % acetic acid overnight [18].

### Assay of hemagglutinating activity

Using a Corning^®^ Costar^®^ 96-Well U-plate (Code: CLS3799; Sigma-Aldrich), twofold serial dilutions of a 50-µL protein sample in phosphate-buffered saline (PBS) were prepared. The same volume of 2 % rabbit red blood cell suspension was added to the wells. After approximately 1 h, the red blood cells in the control (without protein sample) sank to the bottom of the well and appeared as a red spot. The presence of proteins with hemagglutinating activity would cause the cells to clump together and form a plaque [17].

### Carbohydrate specificity test

Twofold serial dilutions of a 50-µL protein sample in PBS were prepared in a 96-well U-plate, and different carbohydrate solutions (glucose, mannose, galactose, glucosamine, maltose, rhamnose, arabinose, *N*-acetylgalactosamine, α-methyl-d-glucoside, α-methyl-pyranoside, raffinose, mannitol, xylitol) were added. The same volume of 2 % rabbit red blood cell suspension was added to the wells. A reduction in hemagglutinating activity indicated that the carbohydrate was specific in its interaction with the lectin, resulting in competitive inhibition of lectin binding with the red blood cells [17].

### Mass spectrometry

Purified lectin was subjected to SDS-PAGE in a polyacrylamide gel (15 % running gel; 5 % stacking gel) at a constant voltage of 120 V for 70 min. The gel was stained, and the area containing the protein band was cut into small pieces. The gel was destained, dehydrated in acetonitrile, and air-dried. A minimal amount of trypsin was added to the gel and incubated at 37 °C for 1 h to allow digestion. The solution containing the tryptic fragments was transferred to a tube, and sent to the laboratory in the Prince of Wales Hospital, Hong Kong, for mass spectrometry analysis according to a previously described procedure [19].

### N-terminal amino acid sequencing

Purified lectin was subjected to SDS-PAGE in a polyacrylamide gel (15 % running gel; 5 % stacking gel) at a constant voltage of 120 V for 70 min, and then transferred onto a polyvinylidene difluoride (PVDF) membrane using a Trans-Blot^®^ SD Semi-Dry Electrophoretic Transfer Cell (Bio-Rad) at a constant voltage of 15 V for 40 min. The membrane was sent to the central laboratory of Beijing Agricultural University, Beijing, for N-terminal amino acid sequencing using a previously described procedure [20].

### Comparison of amino acid sequences

An NCBI BLAST (Standard Protein BLAST: http://blast.ncbi.nlm.nih.gov/Blast.cgi?PROGRAM=blastp&PAGE_TYPE=BlastSearch&LINK_LOC=blasthome) analysis was conducted using the results from the mass spectrometry and N-terminal amino acid sequencing to search for known proteins with similarity to GSLL. The proteins showing the highest scores (most closely related to GSLL) were marked, and the differences in their amino acid sequences were identified.

### Biological activity tests

#### MTT assay

Human nasopharyngeal carcinoma CNE1 and CNE2 cells were purchased from the Sun Yat-sen University of Medicinal Sciences (China). The cells were cultured in Dulbecco’s modified Eagle’s medium containing 10 % fetal bovine serum and 100 U/mL penicillin and streptomycin, and maintained at 37 °C in a humidified incubator under 5 % CO_2_. The cells were seeded onto Corning^®^ Costar^®^ 96-Well plates (Code: CLS3595; Sigma-Aldrich) overnight. Different concentrations of GSLL were added to the cells and incubated at 37 °C for 24, 48, or 72 h. The effects of addition of 62.5 mM glucose or mannose on the GSLL treatment of CNE1 cells were examined to investigate the inhibitory effects of the carbohydrates on the action of GSLL on CNE1 cells. Briefly, after the wells were washed with PBS, 25 µL of MTT (5 mg/mL) in PBS was added and incubated for 4 h. Next, 150 µL of DMSO was added to the wells, and the optical density at 580 nm was recorded with a SpectraMax i3 Multi-mode Plate Reader (Molecular Devices, USA). The IC_50_ values for GSLL treatment of the cells were determined as the GSLL concentrations causing 50 % inhibition of the cells.

#### Flow cytometry

CNE1 cells were seeded onto 6-well plates overnight. Different concentrations of GSLL were added to the cells and incubated for 48 h. The cells were then trypsinized and washed with PBS. For assessment of phosphatidylserine externalization, 250 µL of binding buffer containing 1.25 µL of Annexin V-FITC and 0.5 µL of propidium iodide (PI) was added to the cells, followed by incubation in the dark for 15 min. For assessment of mitochondrial depolarization, 250 µL of PBS containing 2.5 µg/mL JC-1 was added to the cells, and incubated in the dark for 15 min. For cell cycle analysis, the cells were fixed in 70 % ethanol at −20 °C for 2 h and washed with PBS, followed by addition of 250 µL PBS containing 5 µL of PI and incubation in the dark for 15 min. The cells were examined with a BD LSRFortessa™ Cell Analyzer (BD Biosciences, USA). The data were analyzed using BD FACSDiva 7.0 software (BD Biosciences).

#### Western blotting

CNE1 cells were seeded onto 90-mm petri dishes and treated with different concentrations of GSLL for 48 h. The cells were trypsinized, washed with PBS, and lysed with RIPA buffer on ice for 2 h. The cell lysates were centrifuged at 20,000×*g* and 4 °C for 30 min, and the supernatants were collected as the protein fractions. SDS-PAGE was performed, followed by semi-dry transfer onto a PVDF membrane using a Trans-Blot^®^ SD Semi-Dry Transfer Cell (Bio-Rad) at a constant voltage of 15 V for 40 min. The PVDF membrane was blocked with 5 % milk in Tris-buffered saline containing 0.1 % Tween-20 (TBST) for 1 h, incubated with a primary antibody (1:1000 in 5 % milk in TBST) at 4 °C overnight, washed with TBST, and incubated with the corresponding horseradish peroxidase (HRP)-associated secondary antibody (1:1000 in 5 % milk in TBST) at room temperature for 2 h. Primary antibodies used included: Rabbit anti-caspase 3 antibody (Code: #9662; Cell Signaling); Rabbit anti-cleaved caspase 8 antibody (Code: #9496; Cell Signaling); Rabbit anti-caspase 9 antibody (Code: #9502; Cell Signaling). Secondary antibody used included HRP-linked anti-rabbit antibody (Code: #7074; Cell Signaling). The presence of the target proteins was visualized with an electrochemiluminescence detection system using an Amersham ECL Western Blotting Detection Kit (Code: RPN2018; GE Healthcare).

### Statistical analysis

Cell culture experiments were performed three times. The percentage inhibition obtained in each trial, and the mean percentage inhibition and standard deviation of the three trials were obtained using Microsoft Office Excel 2003 (Microsoft, USA). *P*-values for each data point were calculated by a two-tailed Student’s *t* test using SPSS 16.0 (SPSS Inc., USA). Data points with *P*-values below 0.05 were determined as significant results.

## Results

**Fig. 1:**
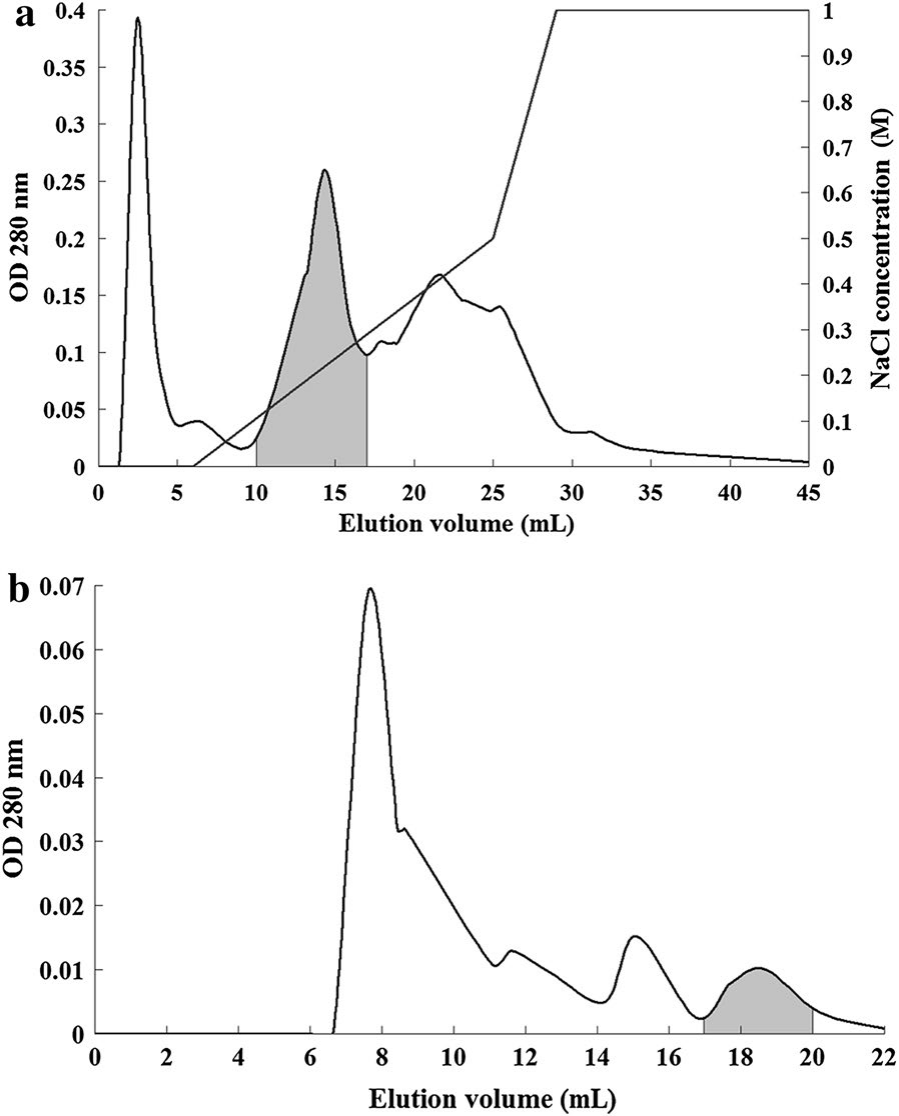
Elution profile for purification of GSLL through **a** Mono Q and **b** Superdex 75

**Fig. 2:**
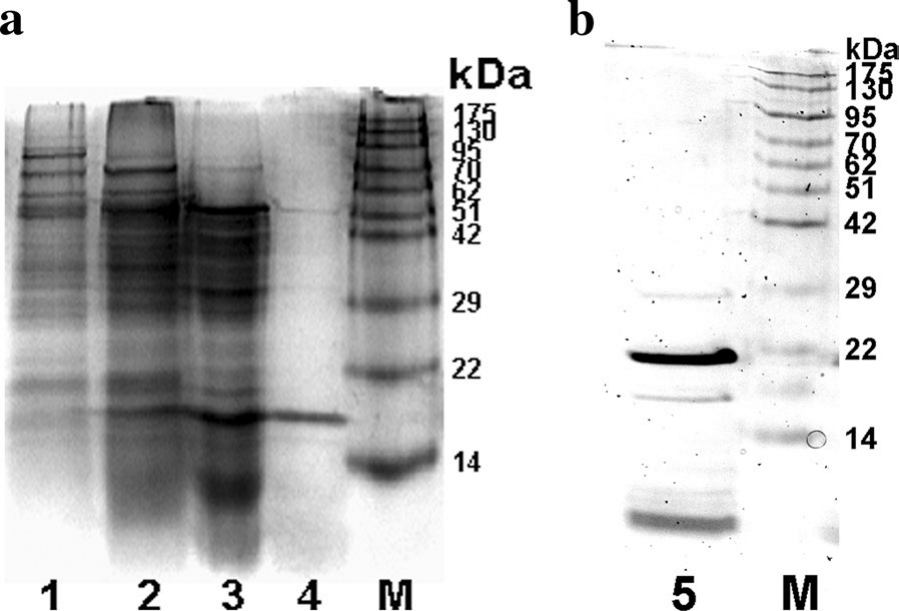
**a** Results of reducing SDS-PAGE of fractions from various steps of purification using a 15 % polyacrylamide gel. *Lane 1* crude seed extract; *lane 2* Affi-gel blue gel, bound fraction; *lane 3* Mono Q, first bound peak, purified lectin from Superdex 75, last peak; *lane M* protein ladder. **b** Results of non-reducing SDS-PAGE of GSLL using a 15 % polyacrylamide tricine gel. *Lane 5* GSLL; *lane M* protein ladder

The current protocol used for purification of GSLL from green speckled lentil seeds comprised ion exchange chromatography (SP-Sepharose: cation exchanger; Mono Q: anion exchanger), affinity chromatography (Affi-gel blue gel), and size exclusion chromatography (Superdex 75). After the first two chromatographic steps (non-adsorption on SP-Sepharose and adsorption on Affi-gel blue gel), GSLL was purified by about 5.6-fold (Table 1). A major absorbance peak after this purification process was detected in the unbound fractions, and two peaks were detected in the bound fractions eluted with a gradient of increasing NaCl concentrations (Fig. 1a). The first bound peak (shaded in grey) represented the bulk of GSLL, which was collected from the final FPLC-gel filtration step on the Superdex 75 column. One major and three minor absorbance peaks were observed (Fig. 1b). The last minor peak constituted purified GSLL. From 80 g of lentil seeds, approximately 11.8 mg of GSLL was isolated, accounting for about 13 % of the total activity. Using this protocol, approximately 50-fold purification of GSLL was achieved. The efficiency of GSLL purification in each step could be observed by the SDS-PAGE results (Fig. 2a). In reducing SDS-PAGE using a 15 % polyacrylamide gel, the purified GSLL obtained from Superdex 75 yielded a 17-kDa band (Fig. 2a). When GSLL was subjected to non-reducing SDS-PAGE using a 15 % polyacrylamide tricine gel, a sharp 21-kDa band with two smaller lighter bands (17 and ~4 kDa) were observed (Fig. 2b). Hence, GSLL consisted of two subunits represented by these two smaller bands.

**Table 1:** Steps for purification of GSLL

Purification step	Yield (mg)/80 g seeds	Specific hemagglutinating activity (units/mg)	Total hemagglutinating activity (10^5^ units)	Recovery of hemagglutinating activity (%)	Fold of purification
Crude extract	4647	138	6.42	100	1
Affi-gel blue gel	580.9	768	4.46	69.4	5.57
Mono Q	124.9	1024	1.28	19.9	7.42
Superdex 75	11.8	6960	0.82	12.8	50.43

Both ion exchange chromatography steps were carried out in a pH 7.6 buffer. GSLL was adsorbed on Mono Q, but not on SP-Sepharose, indicating that GSLL was anionic at this pH, and had a pI value lower than pH 7.6. However, GSLL was eluted from Superdex 75 at about 18.5 mL. Based on the Superdex 75 calibration curve for size determination, GSLL should have a molecular size of approximately 2.6 kDa. The actual size of GSLL should be much larger (≥21 kDa) than the observed size, meaning that GSLL was not eluted from Superdex 75 in keeping with its molecular size.

Mass spectrometry after trypsin digestion (cutting of lysine and arginine residues on the C-terminal side unless the next amino acid was proline) of GSLL was performed, and the mass values obtained were searched using NCBI BLAST to find matching peptides. In the NCBI BLAST analysis, GSLL showed the highest similarity to LcL with 275 amino acids (Table 2). Nine fragments of GSLL were found to match, covering 29 % of the sequence of LcL.

**Table 2:** Results for mass spectrometry of trypsin-digested GSLL

No.	Sequence				
**1**	MASLQTQMIS	FYLIFLSILL	TTIFFFKVNS	TETTSFSITK	**FSPDQK**NLIF
**51**	QGDGYTTKGK	LTLTK**AVKST**	**VGRALYSTPI**	**HIWDR**DTGNV	ANFVTSFTFV
**101**	IDAPSSYNVA	DGFTFFIAPV	DTKPQTGGGY	LGVFNSK**EYD**	**KTSQTVAVEF**
**151**	**DTFYNAAWDP**	**SNKERHIGID**	**VNSIK**SVNTK	**SWNLQNGER**A	NVVIAFNAAT
**201**	NVLTVTLTYP	NSLEEENVTS	YTLNEVVPLK	**DVVPEWVR**IG	FSATTGAEFA
**251**	AHEVHSWSFH	SELGGTSSSK	QAADA		

The results were matched and compared using NCBI BLAST, and GSLL showed highest similarity to LcL

Match to: **LEC_LENCC (LcL)** Score: **57**

Nominal mass (Mr): **30261**; Calculated pI value: **5.37**

Number of mass values searched: **90**; Number of mass values matched: **9**; Sequence Coverage: **29** **%**

Matched sequence no.: **41–46**, **66–68**, **69–73**, **74–85**, **138–141**, **142–163**, **164–175, 181–189**, **231–238**; Matched peptides shown in **boldface**

The sequence of the first 15 amino acids from the N-terminus of GSLL was found to be TETTSFSITKFSPDQ. This sequence was identical to amino acids 31 through 45 of LcL, as shown in Table 2. A protein BLAST search on this segment revealed that it matched with LcL (100 % identical), as well as some other species (e.g., *Lens nigricans* [100 % identical], *Cajanus cajan* [100 % identical], *Vigna aconitifolia* [100 % identical], *Cicer arietinum* [100 % identical], *Lathyrus ochrus* [93 % identical], *Pisum sativum* [93 % identical], *Lathyrus sativus* [93 % identical]) (Table 3).

**Table 3:** Protein BLAST results for the N-terminal amino acid sequence of GSLL

Description		Sequence		Score	Identity (%)
GSLL	1	TETTSFSITKFSPDQ	15		
Lentil lectin Chain A	1	TETTSFSITKFSPDQ	15	50.3	100
Lentil lectin precursor	31	TETTSFSITKFSPDQ	45	50.3	100
lectin [*Lens nigricans*]	31	TETTSFSITKFSPDQ	45	50.3	100
lectin [*Cajanus cajan*]	31	TETTSFSITKFSPDQ	45	50.3	100
lectin [*Vigna aconitifolia*]	24	TETTSFSITKFSPDQ	38	50.3	100
lectin [*Cicer arietinum*]	24	TETTSFSITKFSPDQ	38	50.3	100
Isolectin [*Lathyrus ochrus*-Chain A]	1	TETTSFSITKF**G**PDQ	15	46.9	93
Lectin [Garden Pea (*Pisum Sativum*)]	1	TETTSF**L**ITKFSPDQ	15	44.3	93
lectin [*Lathyrus sativus*]	22	TETTSF**L**ITKFSPDQ	36	44.3	93

Residues that differ from GSLL are in boldface and underlined

**Fig. 3:**
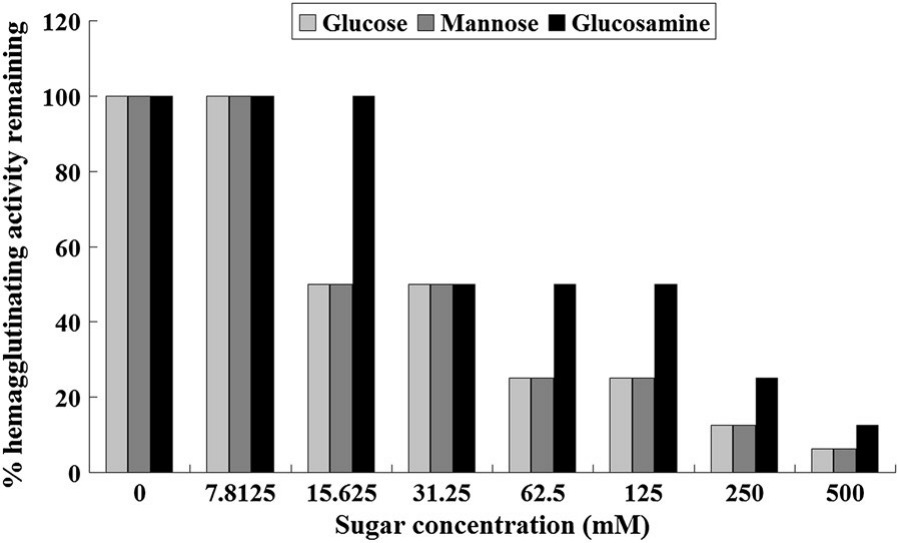
Effects of glucose, mannose, and glucosamine on the hemagglutinating activity of GSLL. Increases in the concentrations of the specific sugars of GSLL strengthened the effects of competitive inhibition on the hemagglutinating activity. The tests were performed three times

In the carbohydrate specificity test, addition of specific binding sugars for GSLL would lead to competition for binding sites of GSLL, causing competitive inhibition of GSLL binding onto the surface sugars of the red blood cells, thereby reducing the hemagglutinating activity of GSLL. The results revealed that GSLL was a glucose- and mannose-specific lectin. Among the various tested carbohydrates, galactose, rhamnose, arabinose, N-acetylgalactosamine, raffinose, mannitol, and xylitol did not affect the hemagglutinating activity of GSLL. On the other hand, glucose and mannose strongly inhibited the hemagglutinating activity of GSLL, followed by glucosamine, while maltose, α-methyl-d-glucoside, and α-methyl-pyranoside slightly inhibited the hemagglutinating activity. The minimal concentrations of glucose and mannose with an inhibitory effect were about 15.6 mM, and that of glucosamine was about 31.2 mM (Fig. 3). Moreover, increases in the sugar concentrations enhanced the inhibitory effect, and the residual hemagglutinating activity was further reduced.

**Fig. 4:**
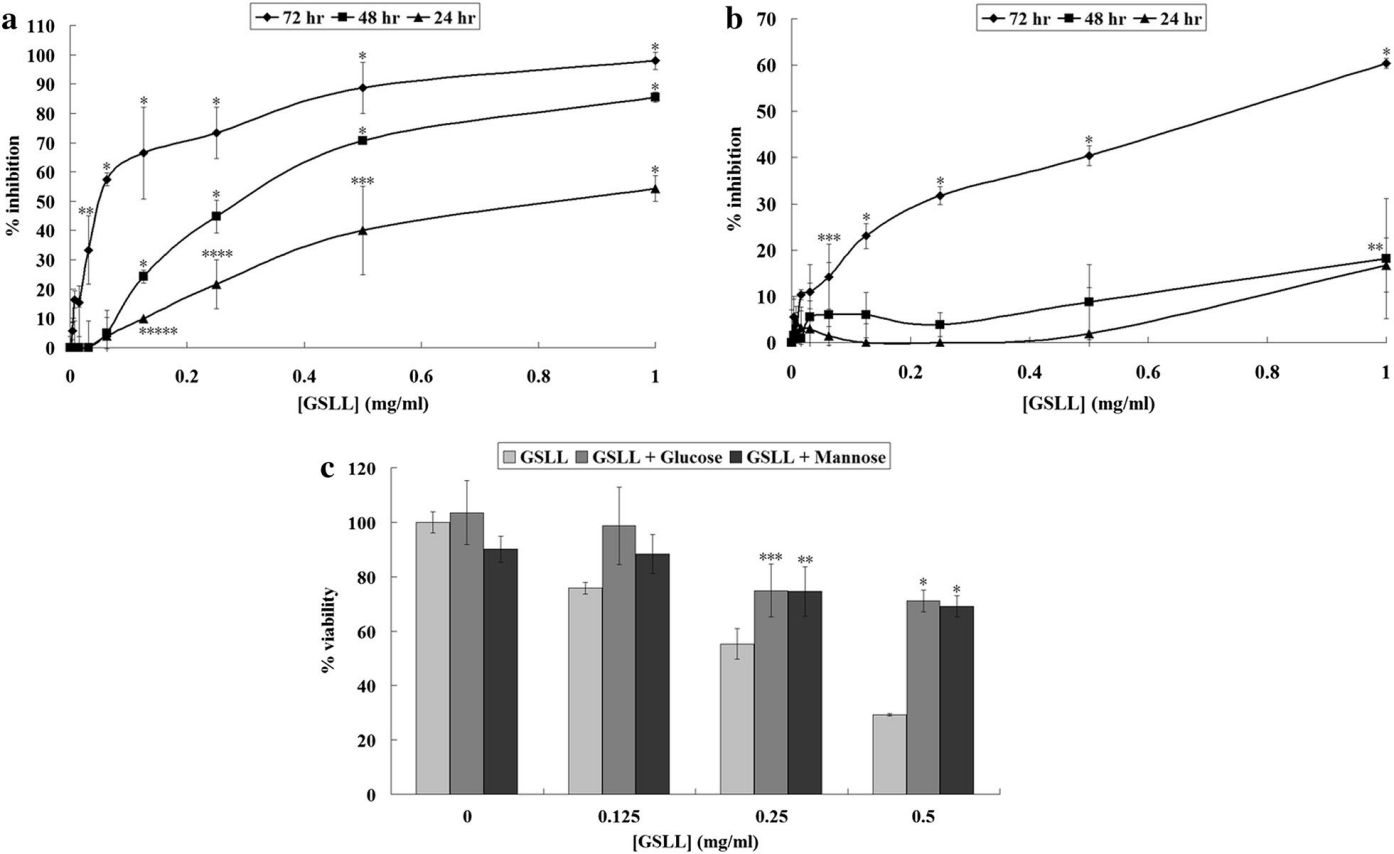
Results of the MTT assay for GSLL-treated **a** CNE1 and **b** CNE2 cells for 24, 48, or 72 h, and **c** GSLL-treated CNE1 cells under co-treatment with 62.5 mM glucose or mannose for 48 h. Data represent mean ± SD (*n* = 3). The *marked data points* represent those with significant differences of *P* < 0.05: **a** *P < 0.001, **P = 0.004, ***P = 0.0082, ****P = 0.0095, *****P = 0.0113; **b** *P < 0.001, **P = 0.004, ***P = 0.044; **c** *P < 0.001, **P = 0.036, ***P = 0.043

In the MTT assay, GSLL inhibited cell proliferation. GSLL strongly inhibited nasopharyngeal carcinoma CNE1 cells, and slightly inhibited CNE2 cells. Treatment with 0–1 mg/mL GSLL resulted in antiproliferative effects on CNE1 cells. After treatment with GSLL for 24, 48, and 72 h, significant inhibition was observed for the GSLL concentrations of 0.125 mg/mL (*P* = 0.0113), 0.125 mg/mL (*P* < 0.001), and 0.031 mg/mL (*P* = 0.004), respectively, and their respective IC_50_ values were 0.95, 0.28, and 0.06 mg/mL (Fig. 4a). CNE2 cells were much less responsive to GSLL treatment. Non-significant antiproliferative activity was detected after 24 h (i.e. for 1 mg/mL GSLL treatment, the data point with 16 % inhibition was *P* > 0.05). Slight inhibition of 18 % was detected after treatment with 1 mg/mL GSLL for 48 h (*P* = 0.004). The activity was more obvious when the duration of GSLL treatment was lengthened to 72 h, and the inhibitory effect was significant starting from 0.063 mg/mL GSLL (*P* = 0.044), with an IC_50_ value of 0.75 mg/mL (Fig. 4b). In the presence of 62.5 mM glucose or mannose, the specific sugars of GSLL, the antiproliferative effects of GSLL on CNE1 cells were reduced (Fig. 4c). After treatment with 0.25 mg/mL GSLL for 48 h, the presence of glucose and mannose significantly reduced the inhibitory effects of the lectin on CNE1 cells from 55.25 to 74.85 % (*P* = 0.043) and 74.52 % (*P* = 0.036), respectively. After treatment with 0.5 mg/mL GSLL, the inhibitory effects were reduced from 29.25 to 71.05 % (*P* < 0.001) and 69.11 % (*P* < 0.001), respectively, in the presence of glucose and mannose. The IC_50_ values after GSLL treatment were also elevated from 0.28 to 1.11 mg/mL and 1.79 mg/mL with the addition of glucose and mannose, respectively.

**Fig. 5:**
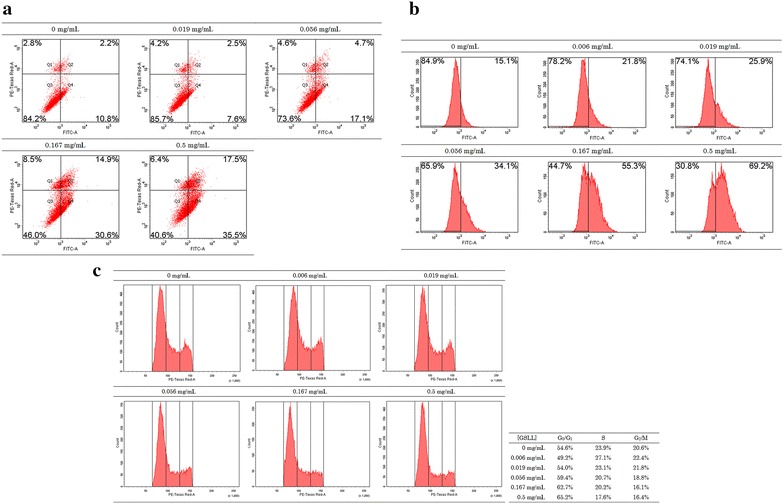
Results of flow cytometry for 48-h GSLL-treated CNE1 cells examined for **a** phosphatidylserine externalization (with annexin V-FITC and PI staining), **b** mitochondrial depolarization (with JC-1 staining), and **c** cell cycle analysis (with PI staining)

**Fig. 6:**
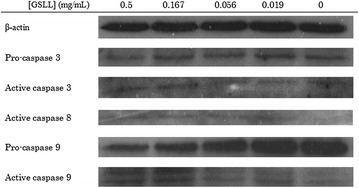
Results of western blotting analysis for 48-h GSLL-treated CNE1 cells

In flow cytometry experiments, GSLL induced apoptosis in CNE1 cells. Upon annexin V-FITC and PI staining, an increase in GSLL concentration from 0 to 0.5 mg/mL led to a rightward shift of the majority of the cells, the population of cells located at the lower left quadrant (healthy cells) was decreased, and the population located at the lower right quadrant (early apoptotic cells with phosphatidylserine externalization) was increased. The cell population at the upper right quadrant (late apoptotic or necrotic cells) was also increased, indicating that GSLL was cytotoxic toward the cells (Fig. 5a). Upon JC-1 staining, an increase in GSLL concentration from 0 to 0.5 mg/mL led to an increase in the intensity of green fluorescence (cells with mitochondrial depolarization) in the majority of the cells (Fig. 5b). In cell cycle analysis, an increase in GSLL concentration from 0 to 0.5 mg/mL reduced the populations of cells at S phase and G_2_/M phase (Fig. 5c), indicating that GSLL blocked cell cycle progression by arresting the cells at G_1_ phase. From the western blotting analysis, GSLL treatment triggered apoptosis of CNE1 cells with activation of caspase 3, 8, and 9 (Fig. 6). Specifically, an obvious decrease in the level of pro-caspase 9 and a slight decrease in pro-caspase 3 were detected, while the levels of active cleaved caspase 3 and 8 were slightly elevated, and that of active caspase 9 was more significantly increased.

## Discussion

The sequence of GSLL partially matched that of LcL. LcL has a 30-kDa precursor form with 275 amino acids; the 1st to 30th amino acids form the signal peptide chain, the 31st to 210th amino acids belong to the lectin β-chain, and the 218th to 269th amino acids belong to the lectin α-chain, with the remaining amino acids located in the propeptides. LcL is a heterotetramer composed of two α-chains and two β-chains [8, 9]. The two chains have isoelectric points of pH 8.2 and 8.8, respectively, and that of their native form is pH 8.6 [21].

There were dissimilarities of GSLL from LcL. In the purification protocol, GSLL was not adsorbed on the cation exchanger SP-Sepharose, but was adsorbed on the anion exchanger Mono Q in pH 7.6 Tris–HCl buffer. GSLL should have a pI value below 7.6, which was lower than that of LcL [21]. The matching sequences of GSLL mainly fell into the β-chain of LcL, covering 73 of 180 amino acids of the β-chain (40.6 %). A segment of GSLL also matched with the α-chain of LcL, covering 8 of 50 amino acids of the α-chain (16 %). GSLL appeared as a 21-kDa band in non-reducing SDS-PAGE, with subunits displaying molecular sizes of 17 and ~4 kDa. The size of the 17-kDa band corresponded with that of the β-chain of LcL. However, the ~4-kDa subunit of GSLL was smaller than the α-chain of LcL (5.7 kDa) [8]. While the N-terminal amino acid sequence of GSLL was found to be TETTSFSITKFSPDQ, which was identical to that of the LcL β-chain, GSLL probably had at least 83 amino acids matching with the 180 amino acids of the LcL β-chain (46.1 %).

From the results of the western blotting analysis, GSLL increased the activation of caspase 3, 8, and 9 in CNE1 cells, suggesting an initiation of the caspase cascade in apoptosis.

LcLs can bind to cell surface polysaccharides and glycoproteins [22, 23]. GSLL showed a strong inhibitory effect on CNE1 cells, but exhibited a slight suppressive action on CNE2 cells. CNE1 is a well-differentiated nasopharyngeal carcinoma cell line, while CNE2 is a poorly-differentiated nasopharyngeal carcinoma cell line [24]. These two nasopharyngeal carcinoma cell lines exhibit differences in morphology, and expression levels of surface proteins. Despite its similar carbohydrate specificity with LcL, GSLL showed differential binding capability to these cell lines.

GSLL exhibited cytotoxic effects on the two cell lines. GSLL treatment caused phosphatidylserine externalization, mitochondrial depolarization, and cell cycle arrest, finally leading to cell death. GSLL might induce apoptosis selectively in cells with high expression levels of glucose- and mannose-containing glycoproteins, depending on its carbohydrate binding capability.

There are no previous reports on the induction of apoptosis in tumor cells by lectins from *L. culinaris*. Furthermore, most of the lectins (e.g., *L. nigricans* lectin, *C. cajan* lectin, *V. aconitifolia* lectin) with N-terminal amino acid sequences similar to GSLL have not been reported to elicit apoptosis in tumor cell lines or exhibit antiproliferative activities. To our knowledge, GSLL is the first lectin from the *Lens* genus to exhibit proapoptotic activities on tumor cells.

Among the lectins with N-terminal amino acid sequence homology to GSLL, only *P. sativum* lectin (pea lectin) triggered apoptosis in tumor cells [25]. Similar to GSLL, pea lectin was specific in glucose and mannose binding. It was also composed of two subunits with different molecular sizes, although the sizes of both subunits were slightly larger than those of GSLL (19.5 and 5 kDa). Pea lectin caused 84 % inhibition of Ehrlich ascites carcinoma (EAC) cells at a concentration of 120 µg/mL [25]. Addition of a caspase-3 inhibitor and caspase-8 inhibitor could reduce the inhibitory activity of pea lectin on EAC cells, indicating an association of this activity with the extrinsic apoptotic pathway involving caspase 3, 8, and 9 [25]. GSLL blocked cell cycle progression by arresting the cells at G_1_ phase (Fig. 5c), while pea lectin inhibited EAC cell proliferation by arresting the cells at G_2_/M phase [25].

## Conclusion

GSLL possessed some different properties from LcL (e.g., lower pI), and increased caspase 3, 8, and 9 activity in CNE1 cells.

## Abbreviations

DMSO: dimethyl sulphoxide
ECL: electrochemiluminescence
FPLC: fast protein liquid chromatography
GSLL: green speckled lentil lectin
HRP: horseradish peroxidase
LcH: *Lens culnaris* hemagglutinin
LcL: *Lens culnaris* lectin
MTT: 4,5-dimethylthiazol-2-yl)-2,5-diphenyltetrazolium bromide
PBS: phosphate buffered saline
PI: propidium iodide
PVDF: polyvinylidene difluoride
SDS-PAGE: sodium dodecyl sulphate-polyacylamide gel electrophoresis
